# A Comparison of Treatment Options for Right Ventricular Outflow Tract Obstruction

**DOI:** 10.1055/a-2796-6906

**Published:** 2026-02-27

**Authors:** Sarah Simona Rahlfs, Jörg Siegmar Sachweh, Rainer Gerhard Kozlik-Feldmann, Daniel Biermann, Michael Hübler, Henning Carstens

**Affiliations:** 1 Klinik und Poliklinik für Kinderherzmedizin und Erwachsene mit angeborenen Herzfehlern, Universitätsklinikum Hamburg-Eppendorf, Hamburg, HH, Germany; 2 Abteilung für Interdisziplinäres Kinderherzprogramm, Universitätsklinikum Hamburg-Eppendorf Universitäres Herzzentrum Hamburg GmbH, Hamburg, German; 3 Department of Surgery for Congenital Heart Disease, University Heart and Vascular Center Hamburg, Hamburg, Germany; 4 Universitätsklinikum Hamburg-Eppendorf, Hamburg, HH, Germany

**Keywords:** pediatric, pulmonary valve, congenital heart disease, CHD, ToF, Fallot, Monocusp

## Abstract

**Background:**

Right ventricular outflow tract (RVOT) obstruction is a hallmark of tetralogy of Fallot (ToF) and related anatomies. Surgical strategies include valve-sparing techniques or transannular patch (TAP) enlargements, optionally with monocusp patch plasty to restore valve competence. This study compares short- and medium-term outcomes of these strategies based on institutional data.

**Methods:**

A retrospective analysis was conducted on 83 ToF patients who underwent surgery between 2007 and 2021. Median age and weight at surgery were 164 days and 6.0 kg, respectively. Patients were grouped by surgical approach: valve-sparing (commissurotomy/delamination, *n* = 27; primary infundibulotomy, *n* = 7) and TAP (without monocusp, *n* = 38; with monocusp, *n* = 11). The primary endpoint was freedom from reintervention.

**Results:**

Significant preoperative differences were found between groups, including valve morphology, pulmonary annulus z-values, oxygen saturation, and prior palliative interventions. Valve-sparing techniques were associated with less postoperative moderate/severe pulmonary regurgitation (17.6% versus 73.5%; *p* < 0.001) and a trend toward shorter ICU stays. TAP with monocusp resulted in significantly less pulmonary regurgitation than TAP alone (36.4% versus 84.2%; *p* = 0.002) and showed a trend toward fewer reinterventions after 5 years (0% versus 38.5%; *p* = 0.073), with a significant difference at 10 years (14.3% versus 71.4%; *p* = 0.024).

**Conclusion:**

Valve-sparing approaches yield better early outcomes and fewer long-term reinterventions when anatomically feasible. When TAP is necessary, adding a monocusp patch significantly reduces postoperative regurgitation and improves long-term durability.

## Introduction

Surgical repair of Tetralogy of Fallot (ToF) has become a standard procedure with low mortality. 1 Optimal relief of right ventricular outflow tract obstruction (RVOTO) is a fundamental component of surgical treatment for patients with ToF and Fallot-like anatomies. In particular, the best strategies for treating valvular stenosis with borderline pulmonary valve annulus sizes are the subject of clinical research. 2 3 Depending on the size and morphology of the valve, RVOTO at the level of the pulmonary valve can be treated with valve-sparing techniques, such as commissurotomy or delamination plasty. 4 In instances where a morphological substrate for reconstruction is absent, particularly in cases of severe pulmonary annular hypoplasia, inadequate pulmonary leaflet tissue quality, and deficient pulmonary valve function, reconstruction surgery often fails to yield a satisfactory outcome. In such cases, transvalvular enlargement using a transannular patch (TAP) is often the only viable option for adequately mitigating the stenosis. Consequently, this results in a loss of valve function, which frequently leads to severe pulmonary valve (PV) regurgitation. This, in turn, can subsequently lead to right ventricular (RV) dilatation and ventricular arrhythmias. 5 6 A variety of surgical techniques have been described as methods to restore valve function despite valvular transection, including the use of newly created monocusp pulmonary valves (MPVs) into the TAP. 6 7 The concept of restoring pulmonary valve function by implanting a patch that functions as a monocuspid valve was first described by W. Lillehei in 1964. The MPV can be made from human, xenogeneic, or synthetic materials. 6 It is sutured into the area of the opened valve ring and connected to the TAP. 8 However, this procedure is more complex and time-consuming. The result of the valve function is difficult to assess intraoperatively and can therefore often only be determined in postoperative echocardiography. Furthermore, it was found to have poorer long-term results than valve-sparing procedures. 9 Although this approach may appear to be reasonable at first glance, the results of the MPV technique have proven to be inconsistent, particularly with regard to valve function. 9 10 11 12 13 14 The objective of this study was to examine the results obtained at our center in the surgical treatment of RVOTO in patients with typical ToF anatomy, with the aim of potentially deriving concepts for treatment options in cases with borderline pulmonary valve size or conformity.

## Material and Methods

### Patients

This retrospective cohort analysis included 83 consecutive patients with typical ToF anatomy who underwent surgical correction between 2007 and 2021. Baseline demographic and clinical characteristics are summarized in Table 1 . Patients were excluded if pulmonary atresia was present, if a right ventricle–pulmonary artery (RV-PA) conduit had been implanted, if univentricular palliation was performed, or if definitive repair occurred after the age of 2 years.

**Table 1:** Patient characteristics

Variables	Patients ( *n* = 83)
*Anatomy*	
ToF	56 (67%)
DORV/ToF	27 (33%)
Male sex	49 (59%)
*Genetic syndrome*	
No syndrome	61 (74%)
Trisomy 18 or 21	5 (6%)
Di George	5 (6%)
Others	12 (14%)
Age at total correction (d)	164 (IQR 37–475)
Weight at total correction (kg)	6.0 (IQR 3.8–9.3)
*Year of surgery*	
2007–2013	39 (47%)
2014–2021	44 (53%)
*Surgical strategy (RVOT)*	
TAP	38 (45%)
MPV	11 (13%)
Valve sparing	34 (42%)
*Previous palliative procedures*	
No procedure	55 (66%)
Surgical (BT shunt/TAP)	18 (22%)
Interventional (balloon dilatation/stent)	8 (10%)
Combined	2 (2%)

Abbreviations: BT-Shunt, Blalock-Taussig-Shunt; DORV, Double Outlet Right Ventricle; MPV, monocusp pulmonary valve; RVOT, right ventricular outflow tract; TAP, transannular patch; ToF, tetralogy of Fallot.

Surgical approaches were categorized based on the primary strategy applied to the right ventricular outflow tract (RVOT) at the time of ventricular septal defect (VSD) closure. Accordingly, procedures were classified as TAP, MPV reconstruction, commissurotomy, or primary infundibulectomy.

### Statistical Analysis

Clinical data were retrospectively extracted from operative reports, hospital discharge summaries, echocardiographic assessments, and additional medical records. Data collection was performed using Microsoft Excel, and statistical analyses were performed with SPSS software. Patients were categorized according to the RVOT surgical technique applied.

**Fig. 1:**
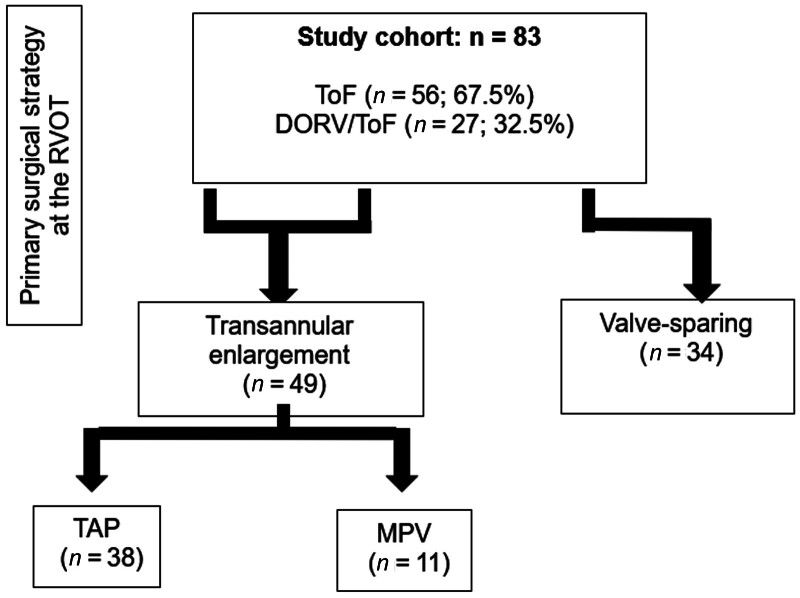
Group division. DORV, double outlet right ventricle; MPV, monocusp pulmonary valve; RVOT, right ventricular outflow tract; TAP, transannular patch; ToF, tetralogy of Fallot.

The primary outcome measure was freedom from reintervention, defined as any surgical or catheter-based procedure involving the RVOT or the pulmonary arteries. Initially, outcomes were compared between patients who underwent annulus-incising procedures (TAP and MPV reconstruction) and those treated with valve-sparing techniques (primary commissurotomy, pulmonary valve annulus delamination, or primary infundibulectomy). Subsequently, a direct comparison between TAP and MPV was performed ( Fig. 1 ).

For analysis purposes, long-term follow-up data were aggregated into two intervals: years 4 to 6 and years 9 to 11 after repair. In cases where a reintervention occurred within these intervals, only echocardiographic data obtained after the intervention were considered. Continuous variables are presented as median values with corresponding ranges. Data distribution was assessed using the Kolmogorov–Smirnov test. Depending on normality, intergroup comparisons were performed using either Student's *t* -test or the Mann–Whitney U test. Categorical variables were analyzed using the chi-square test, with Fisher's exact test applied when expected frequencies were below 5.

To identify potential confounding factors influencing time to reintervention, multivariable Cox proportional hazards regression models were constructed for each of the two primary comparisons. All statistical tests were two-sided, and a *p* -value < 0.05 was considered indicative of statistical significance.

## Results

### Comparison of Valve-sparing and Transannular Enlargement Techniques

**Fig. 2:**
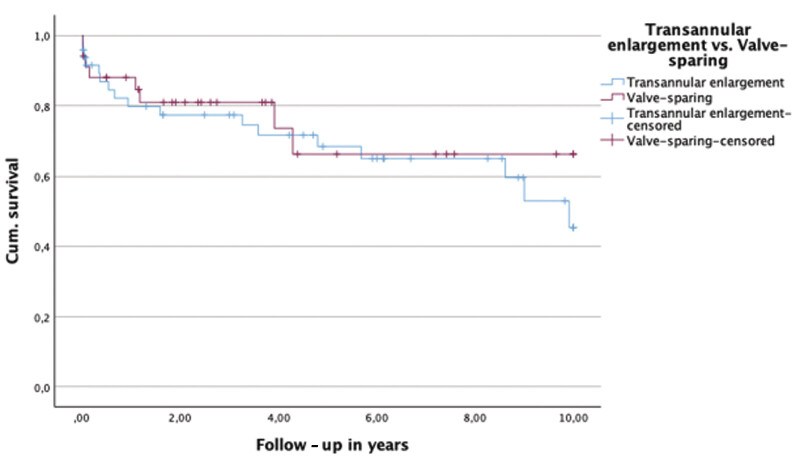
Kaplan-Meier (KM) curve: Freedom from reinterventions—valve-sparing versus enlargement. Log rank: χ ^2^ = 0.321, *p* = 0.571.

Patient baseline characteristics are summarized in Table 2 . Comparison between transannular enlargement and valve-preserving surgical approaches demonstrated no significant differences in demographic parameters, including age, body weight, sex, surgical era (before versus after 2014), or preoperative maximal systolic RVOT gradient. In contrast, patients treated with transannular enlargement more frequently presented with bicuspid pulmonary valve morphology ( *p* = 0.005), smaller pulmonary valve annulus Z-scores ( *p* < 0.001), lower preoperative arterial oxygen saturation ( *p* = 0.015), and a higher prevalence of previous palliative procedures ( *p* < 0.001). Intraoperative variables, including aortic cross-clamp duration, early postoperative residual RVOT gradient, and right ventricular function, did not differ significantly between groups. Postoperative intensive care unit length of stay tended to be shorter following valve-sparing repair, although this did not reach statistical significance (mean 7.29 versus 8.83 days; *p* = 0.066). The incidence of moderate-to-severe pulmonary regurgitation (PR) was substantially higher in the transannular enlargement cohort compared with valve-sparing techniques (73.5% versus 17.6%; *p* < 0.001). Long-term follow-up outcomes are detailed in Table 3 . No significant intergroup differences were identified with respect to right ventricular pressure, RVOT gradient, or tricuspid regurgitation severity. At 5 years postoperatively, severe or free PR was present in 38.7% of patients undergoing transannular enlargement and in 16.7% of those treated with valve preservation. At 10 years, corresponding rates were 28.5 and 0%, respectively. Rates of reintervention did not differ significantly between surgical strategies. Freedom from reintervention is illustrated using a Kaplan-Meier curve in Fig. 2 . Multivariable Cox proportional hazards regression analysis ( Table 4 ) did not identify any clinically relevant confounding variables influencing freedom from reintervention.

**Table 2:** Surgery data

Variables	Transannular enlargement ( *n* = 49)	Valve-sparing ( *n* = 34)	*P* -value	TAP ( *n* = 38)	MPV ( *n* = 11)	P-value
*Demographic data* Male sex Age (d) Weight (kg) Year of surgery 2007–2013 2014–2021	27 (55.1%) 172 (±72) 6.079 (±1.279) 26 (53.1%) 23 (46.9%)	22 (64.7%) 167 (±68) 6.205 (±1.503) 13 (38.2%) 21 (61.8%)	n.s. n.s. n.s. n.s.	22 (57.9%) 172 (±73) 6.148 (±1.363) 20 (52.6%) 18 (47.4%)	5 (45.5%) 174 (±50) 5.842 (±0.946) 6 (54.5%) 5 (45.5%)	n.s. n.s. n.s. n.s.
*Cardiac anatomy and functional data (preoperative)* Pulmonary valve morphology bicuspid a RVOT gradient (mmHg) PV Z-score SpO _2_ (%) Palliative surgical procedures	35 (87.5%); *n* = 40 83.89 (±18.60) −3.99 (±2.56) 91.61 (±7.28) 25 (51.0%)	20 (58.8%) 79.88 (±24.16) −1.41 (±1.87) 95.15 (±6.01) 3 (8.8%)	**0.005** n.s. **<0.001** **0.015** **<0.001**	28 (87.5%); *n* = 32 85.2 (±19.44) −3.87 (±2.66) 91.58 (±7.38) 19 (50%)	7 (87.5%); *n* = 8 79.73 (±15.70) −4.40 (±2.27) 91.73 (±7.30) 6 (54.5%)	n.s. n.s. n.s. n.s. n.s.
*Intraoperative data* ACC time (min)	109 (±32)	104 (±30)	n.s.	111 (±35)	104 (±16)	n.s.
Postoperative ICU stay (d)	8.83 (±8.28)	7.29 (±7.80)	0.066	8.77 (±8.61)	9.00 (±7.51)	n.s.
*Discharge echocardiography* Mean RVOT gradient > 35 mmHg Moderate or severe PR Reduced right ventricular function	7 (15.2%); *n* = 46 36 (73.5%) 9 (18.4%); *n* = 45	9 (31%); *n* = 29 6 (17.6%) 11 (32.4%); *n* = 31	n.s. **<0.001** n.s.	5 (13.9%); *n* = 36 32 (84.2%) 7 (18.4%); *n* = 34	2 (20%); *n* = 10 4 (36.4%) 2 (18.2%); *n* = 11	n.s. **0.002** n.s.

Abbreviations: ACC, aortic cross clamp; MPV, monocusp pulmonary valve; PR, pulmonary regurgitation; PV, pulmonary valve; RVOT, right ventricular outflow tract; TAP, transannular patch.

a One valve monocuspide.

**Table 3:** Follow-up data

Variables	Transannular enlargement	Valve-sparing	*P* -value	TAP	MPV	*P* -value
*5-year follow-up*	*n* = 33	*n* = 14		*n* = 26	*n* = 7	
RV pressure (mmHg)	28.73 (±16.93); *n* = 22	24.39 (±9.95); *n* = 9	n.s.	30.72 (±18.06); *n* =18	19.75 (±4.99); *n* = 4	n.s.
RVOT gradient > 35 mmHg	2 (6.1%)	2 (14.3%)	n.s.	2 (7.7%)	0	n.s.
Severe or free PR	12 (38.7%); *n* = 31	2 (16.7%); *n* = 12	n.s.	9 (37.5%); *n* = 24	3 (42.9%)	n.s.
Moderate or severe TR	3 (9.1%)	0	n.s.	3 (11.5%)	0	n.s.
Patients with reinterventions Number of reinterventions * 1* * >1*	10 (30.3%) 5 (15.1%) 5 (15.1%)	4 (28.6%) 4 (28.6%) 0	n.s. n.s.	10 (38.5%) 5 (19.2%) 5 (19.2%)	0 0 0	n.s. n.s.
*10-year follow-up*	*n* = 21	*n* = 4		*n* = 14	*n* = 7	
RV pressure (mmHg)	31.44 (±20.47); *n* = 16	26.67 (±13.73); *n* = 3	n.s.	33.14 (±24.44); *n* = 11	27.70 (±7.24); *n* = 5	n.s.
Mean RVOT gradient > 35 mmHg	1 (4.8%)	1 (25%)	n.s.	1 (7.1%)	0	n.s.
Severe or free PR	6 (28.5%)	0	n.s.	3 (21.4%)	3 (42.9%)	n.s.
Moderate or severe TR	4 (19.1%)	0	n.s.	3 (21.4%)	1 (14.3%)	n.s.
Patients with reinterventions Number of reinterventions 1 >1	11 (52.4%) 4 (19.0%) 7 (33.3%)	1 (25%) 1 (25%) 0	n.s. n.s.	10 (71.4%) 4 (28.6%) 6 (42.9%)	1 (14.3%) 0 1 (14.3%)	**0.024** n.s.

Abbreviations: MPV, monocusp pulmonary valve; PR, pulmonary regurgitation; RV, right ventricular; RVOT, right ventricular outflow tract; TAP, transannular patch; TR, Tricuspid regurgitaon.

**Table 4:** Multivariate Cox regression: transannular enlargement versus valve-sparing versus MPV

Variables	HR (95% CI) TAP vs. valve-sparing	*P* -value TAP vs. valve-sparing	HR (95% KI) TAP vs. MPV	*P* -value TAP vs. MPV
Surgical procedure	1.336 (0.474; 3.763)	n.s.	0.288 (0.063; 1.307)	n.s.
PV Z-score	0.922 (0.778; 1.092)	n.s.	0.927 (0.765; 1.123)	n.s.
Age at total correction (d)	0.993 (0.985; 1.001)	n.s.	0.991 (0.982; 1.000)	**0.049**
Palliative surgical procedures	2.404 (0.990; 5.836)	n.s.	2.541 (0.877; 7.358)	0.086

Abbreviations: MPV, monocusp pulmonary valve; PV, pulmonary valve; TAP, transannular patch.

### Comparison of Transannular Patch Versus Monocusp Pulmonary Valves Reconstruction

**Fig. 3:**
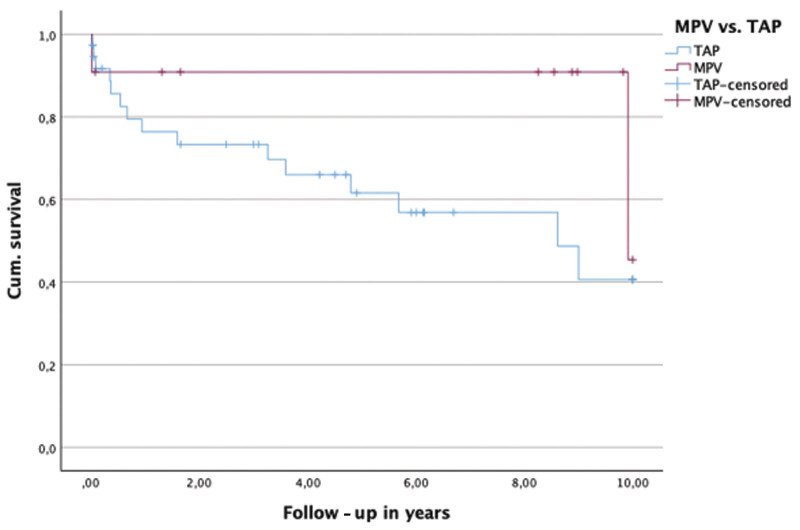
Kaplan-Meier (KM) curve: Freedom from reinterventions transannular patch (TAP) versus monocusp pulmonary valve (MPV). Log rank: χ ^2^ = 1.997, *p* = 0.158.

**Fig. 4:**
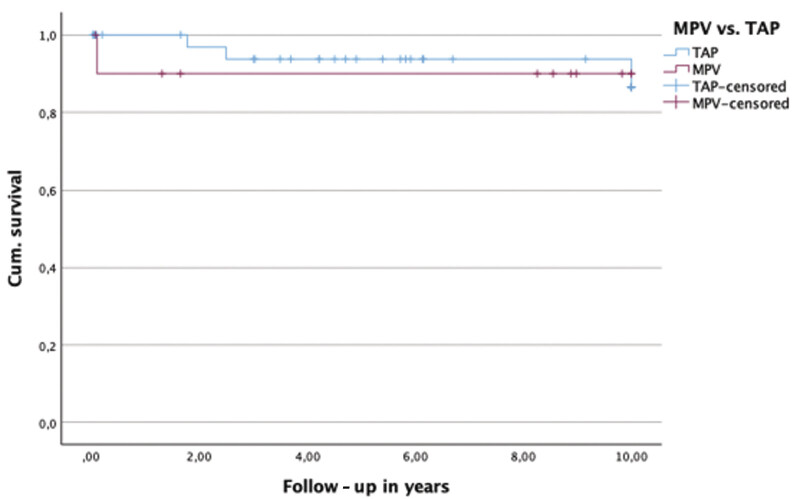
Kaplan-Meier (KM) curve: Survival transannular patch (TAP) versus monocusp pulmonary valve (MPV). Log rank: χ ^2^ = 0.129, *p* = 0.719.

**Fig. 5:**
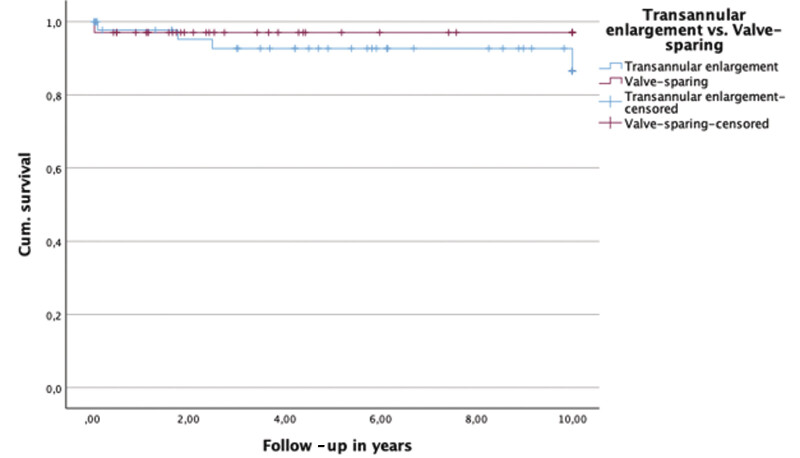
Kaplan-Meier (KM) curve: Survival valve-sparing versus enlargement. Log rank: χ ^2^ = 0.597 *p* = 0.440.

Baseline patient characteristics are presented in Table 2 . No statistically significant differences were observed between the TAP and MPV reconstruction groups with respect to demographic variables (age, body weight, year, sex), cardiac anatomical and functional parameters (pulmonary valve morphology, systolic RVOT gradient, pulmonary valve annulus size, preoperative oxygen saturation, or prior palliative procedures), or perioperative data, including aortic cross-clamp duration, postoperative intensive care unit stay, residual RVOT gradient, and early postoperative right ventricular function. At hospital discharge, the prevalence of moderate-to-severe PR was significantly higher in the TAP cohort compared with the MPV group (84.2% versus 36.4%; *p* = 0.002). Long-term follow-up outcomes are summarized in Table 3 . No significant intergroup differences were identified regarding right ventricular pressure, RVOT gradient, or tricuspid regurgitation severity. At 5 years post-repair, severe or free PR was documented in 37.5% of patients in the TAP group and 42.9% in the MPV group; corresponding proportions at 10 years were 21.4 and 42.9%, respectively. Within the first 5 postoperative years, 38.5% of patients treated with TAP required at least one reintervention, whereas no reinterventions were recorded in the MPV group ( *p* = 0.073). By 10 years of follow-up, reinterventions occurred significantly more frequently in the TAP group than in the MPV group (71.4% versus 14.3%; *p* = 0.024). Freedom from reintervention is illustrated using a Kaplan-Meier curve in Fig. 3 . Functional status at the most recent follow-up is summarized in Table 5 . The majority of patients across all groups (72.7–88.2%) were fully active without limitations in daily activities, while only a small subset (5.3–9.1%) exhibited clinically relevant reductions in functional capacity. No significant differences in functional outcomes were observed between groups. Figs. 4 and 5 compare survival between the TAP and MPV groups and between valve-sparing and transannular enlargement techniques using a Kaplan-Meier curve. Potential confounding variables were explored using Cox proportional hazards regression analysis ( Table 4 ). Only age at the time of primary repair demonstrated a statistically significant association with reintervention risk; however, the observed effect size was minimal (hazard ratio 0.991).

**Table 5:** Survival and endurance

Condition at last follow-up	Transannular enlargement ( *n* = 49)	Valve-sparing ( *n* = 34)	TAP ( *n* = 38)	MPV (n = 11)
Fully functional	38 (77.6%)	30 (88.2%)	30 (78.9%)	8 (72.7%)
Lightly restricted endurance	4 (8.2%)	1 (2.9%)	3 (7.9%)	1 (9.1%)
Restricted endurance	3 (6.1%)	2 (5.9%)	2 (5.3%)	1 (9.1%)
Deceased	4 (8.2%)	1 (2.9%)	3 (7.9%)	1 (9.1%)
	➔ Chi-square: *p* = 0.552		➔ Chi-square: *p* = 0.964	

Abbreviations: MPV, monocusp pulmonary valve; TAP, transannular patch.

## Discussion

The available data demonstrate that surgical treatment of RVOTO at the valvular level in patients with typical ToF anatomy can achieve excellent long-term outcomes using both valve-preserving techniques and transannular enlargement techniques. These results are in line with those reported by other groups. 11 Patients in whom pulmonary valve morphology and annular dimensions allowed for a valve-sparing approach showed a reduced need for reinterventions during follow-up. Residual stenosis, which is accepted or even deliberately maintained with these reconstructive techniques, appears to be hemodynamically better tolerated than the higher-grade pulmonary valve insufficiency associated with transannular enlargement techniques. This concept is further supported by the findings of Blais et al, who evaluated outcomes in 683 patients over a 30-year follow-up period. 14 In their analysis, valve-preserving repair was associated with superior 30-year survival, fewer cardiovascular reinterventions, and a lower rate of pulmonary valve replacement compared with TAP repair, even in the presence of relevant residual pulmonary stenosis. 14 These results are consistent with earlier studies demonstrating that patients undergoing valve-preserving procedures tend to have shorter intensive care unit stays and significantly lower rates of moderate or severe PR than those treated with transannular enlargement. 15 Although PR is often initially well tolerated following surgical correction, it is associated with increased morbidity in the long term. Patients frequently develop impaired exercise capacity as a clinical manifestation of progressive right ventricular dysfunction. Over time, this may lead to right heart failure, the development of cardiac arrhythmias, and, in severe cases, sudden cardiac death. 4 Mortality was lower in the valve-sparing group (2.9%) compared with the TAP group (8.2%). While patients treated with TAP enlargement often required multiple repeat reinterventions during follow-up, no subsequent reinterventions were observed in the valve-sparing cohort. Consequently, there is broad consensus that valve-sparing repair should be preferred over transannular incision with loss of pulmonary valve function whenever this strategy results in only a minor residual stenotic component. In the context of surgical planning, pulmonary valve annulus Z-scores greater than −2, a favorable and non-dysplastic valve morphology, and adequate tissue quality are considered key factors supporting the feasibility of valve preservation. 16 17 In the present study population, the mean pulmonary valve annulus Z-score was −3.99 in the TAP group compared with −1.41 in the valve-sparing group. Furthermore, bicuspid and frequently dysplastic valve morphology was more prevalent in the transannular incision group (87.5%) than in the valve-preserving group (58.8%). This anatomical constellation was also associated with lower preoperative oxygen saturation levels and a more frequent need for systemic-to-pulmonary shunt palliation. Taken together, these findings indicate that, when anatomical conditions permit and adequate relief of RVOTO can be achieved, a valve-preserving surgical approach should be favored.

A subgroup analysis was performed to evaluate whether incorporation of a monocuspid valve into the RVOT, using a technically more demanding surgical approach, is associated with a reduced need for reinterventions in cases requiring transannular enlargement. This analysis was conducted on the basis of the data available in the present cohort. Preoperative comparison of the two groups demonstrated no relevant anatomical or functional differences, underscoring the clinical relevance of the postoperative outcomes for surgical decision-making. Contrary to expectations, no prolongation of aortic cross-clamp time was observed in the monocusp group, likely attributable to the increased technical complexity of the procedure. 18 This finding may be influenced by individual surgeon experience or by concomitant surgical procedures that were not accounted for in the present analysis. Therefore, this observation was considered to be of limited significance. Recent evidence suggests that the use of a monocusp may be associated with reduced postoperative morbidity, primarily due to lower degrees of PR and a consequently less complex postoperative course. 19 In line with this, the present analysis demonstrated a significant reduction in moderate to severe PR in the monocusp group. However, this reduction was not accompanied by a corresponding decrease in intensive care unit stay. This may be explained by the generally favorable tolerability of PR in the early postoperative period, as previously discussed. With regard to long-term outcomes, the existing literature reports heterogeneous results. Several studies have shown that PR also progresses over time in patients with monocusp implantation, although the time to development of severe PR varies considerably. Nevertheless, comprehensive analyses indicate an advantage of the monocusp technique with respect to the need for RVOT reintervention when compared with standard TAP repair. 20 In the present study, freedom from severe PR and reintervention in the monocusp group was 57.1% at 5 years and 42.9% at 10 years, indicating that a substantial proportion of patients did not develop severe PR even during long-term follow-up. In contrast, in the TAP group, the severity of PR appears to play a lesser role in the context of repeated reinterventions. Regarding reintervention rates, the literature presents divergent conclusions. Jang et al hypothesized that monocusp implantation prolongs the interval until pulmonary valve replacement becomes necessary, 20 whereas Park et al reported no clear benefit of the monocusp technique with respect to reintervention rates. 21 In the present cohort, no reinterventions were observed in the monocusp group within the first 5 years, whereas 42.9% of patients in the TAP group required at least one reintervention during this period. After 10 years, this discrepancy became more pronounced, with freedom from reintervention decreasing from 71.4 to 14.3%. These findings suggest that incorporation of a monocuspid valve in surgical strategies requiring transannular enlargement may be associated with a lower rate of subsequent interventions in the present cohort.

## Limitations

This study has several limitations. It is a retrospective, single-center analysis with a relatively small sample size, which inherently limits statistical power. In addition, the reliance primarily on univariate analyses constrains the strength of the conclusions, and the findings should therefore be interpreted cautiously, serving as exploratory rather than definitive evidence. Nevertheless, these limitations do not diminish the study's relevance: it provides rare long-term follow-up in a well-defined cohort and offers valuable insights into the clinical significance of valve preservation. Accordingly, it contributes important evidence to the ongoing discussion on optimal surgical strategies for RVOTO.

## Conclusion

In summary, surgical treatment of RVOTO in patients with a ToF or ToF-like congenital heart defects is associated with excellent long-term results. In cases where it is feasible, it is recommended to prioritize a valve-sparing procedure. In instances where this is not feasible, the current data indicate a potential benefit for monocusp implantation over TAP procedures. This advantage is primarily attributable to a reduced necessity for intervention in the long-term follow-up.
